# Non-dietary factors associated with *n*-3 long-chain PUFA levels in humans – a systematic literature review

**DOI:** 10.1017/S0007114519000138

**Published:** 2019-04-14

**Authors:** Renate H. M. de Groot, Rebecca Emmett, Barbara J. Meyer

**Affiliations:** 1 Welten Institute – Research Centre for Learning, Teaching, and Technology, Open University of the Netherlands, 6419 AT Heerlen, The Netherlands; 2 Department of Complex Genetics, School for Nutrition, Toxicology and Metabolism, Maastricht University, 6200 MD Maastricht, The Netherlands; 3 School of Medicine, Lipid Research Centre, Molecular Horizons, Illawarra Health & Medical Research Institute, University of Wollongong, Wollongong, NSW 2522, Australia

**Keywords:** Fatty acid status, Effects, Determinants, Healthy adults, Review studies, Measurement, Implications

## Abstract

Numerous health benefits are attributed to the *n*-3 long-chain PUFA (*n*-3 LCPUFA); EPA and DHA. A systematic literature review was conducted to investigate factors, other than diet, that are associated with the *n*-3 LCPUFA levels. The inclusion criteria were papers written in English, carried out in adult non-pregnant humans, *n*-3 LCPUFA measured in blood or tissue, data from cross-sectional studies, or baseline data from intervention studies. The search revealed 5076 unique articles of which seventy were included in the qualitative synthesis. Three main groups of factors potentially associated with *n*-3 LCPUFA levels were identified: (1) unmodifiable factors (sex, genetics, age), (2) modifiable factors (body size, physical activity, alcohol, smoking) and (3) bioavailability factors (chemically bound form of supplements, krill oil *v.* fish oil, and conversion of plant-derived *α*-linolenic acid (ALA) to *n*-3 LCPUFA). Results showed that factors positively associated with *n*-3 LCPUFA levels were age, female sex (women younger than 50 years), wine consumption and the TAG form. Factors negatively associated with *n*-3 LCPUFA levels were genetics, BMI (if erythrocyte EPA and DHA levels are <5·6 %) and smoking. The evidence for girth, physical activity and krill oil *v*. fish oil associated with *n*-3 LCPUFA levels is inconclusive. There is also evidence that higher ALA consumption leads to increased levels of EPA but not DHA. In conclusion, sex, age, BMI, alcohol consumption, smoking and the form of *n*-3 LCPUFA are all factors that need to be taken into account in *n*-3 LCPUFA research.

*n*-3 Long-chain PUFA (*n*-3 LCPUFA) are fatty acids with twenty or more carbons, and they are the elongation and desaturation products of the essential fatty acid *α*-linolenic acid (ALA, 18 : 3*n*-3). Whilst there are emerging health benefits of docosapentaenoic acid (DPA, 22 : 5*n*-3)^(^ ^1^ ^)^, the vast majority of health benefits have been attributed to the *n*-3 LCPUFA EPA (20 : 5*n*-3) and DHA (22 : 6*n*-3)^(^ ^2^ ^)^.

*n*-3 LCPUFA have been shown to be important for neurological development in very early pregnancy^(^ ^3^ ^)^, during later pregnancy and lactation^(^ ^4^ ^)^ and cardiovascular health^(^ ^5^ ^,^ ^6^ ^)^ and there is also emerging evidence for mental health^(^ ^7^ ^)^. Several mechanisms have been suggested^(^ ^8^ ^)^, such as their structural role in the cell membrane influencing signal transduction, stimulating neuronal growth, influencing neurotransmitter release and facilitating glucose uptake from the endothelial cells into the brain. *n*-3 LCPUFA are also important precursors of the eicosanoids, resulting in reduced blood clotting and increased blood flow^(^ ^8^ ^)^. DHA is a precursor of docosanoids such as resolvins and maresins, resulting in anti-inflammatory effects^(^ ^9^ ^)^ and neuroprotectins which protect neurons^(^ ^8^ ^)^.

The aforementioned potential health benefits have been observed from a wide variety of evidence including epidemiological, observational studies and randomised controlled trials. However, many studies have failed to measure the *n*-3 LCPUFA in blood or tissue, and this may severely limit the interpretations of the results as these *n*-3 LCPUFA might be influenced by many factors besides intake.

It is well established that diet and supplementation with *n*-3 LCPUFA have the largest impact on *n*-3 LCPUFA levels^(^ ^10^ ^)^; however, research has indicated that factors other than diet also play a role^(^ ^11^ ^)^. As researchers may not be aware of the many non-dietary factors associated with the *n*-3 LCPUFA levels *per se*, and the way these can influence the study outcomes, the aims of the present paper are to (1) report the results of a systematic literature review of the well-described non-dietary factors that are associated with the *n*-3 LCPUFA levels, (2) identify important non-dietary factors that should be considered in future studies and (3) discuss whether measuring *n*-3 LCPUFA levels is necessary in research that assesses the health benefits of *n*-3 LCPUFA.

## Methods

### Search strategy

A Preferred Reporting Items for Systematic Reviews and Meta-Analysis (PRISMA) systematic literature search was conducted using four different electronic databases (ProQuest, Medline, Web of Science and Cochrane). The search was conducted in June 2017 and covered all years up to June 2017. Appropriate truncation and relevant indexing terms were used. Search terms were related to (1) *n*-3 LCPUFA (e.g. *n*-3 fatty acids, EPA, DHA) and (2) factors or determinants associated with/influencing *n*-3 LCPUFA levels (e.g. sex, age, genetics, body size, physical activity, alcohol and smoking). An outline of the search strategy is available in online Supplementary Table S1.

### Comparison of *n*-3 long-chain PUFA levels across studies

For the present review ‘*n*-3 LCPUFA levels’ are used as an umbrella term to describe the *n*-3 fatty acids with twenty or more carbon atoms in any blood or tissue fractions measured. We do not focus on DPA as it appears to have a poor association with diet in epidemiological studies (see Fig. 1(c) in Sullivan *et al.* ^(^ ^12^ ^)^). Please note that various comparable terminologies exist in the literature, including the Holman index; the Lands highly unsaturated fatty acids^(^ ^13^ ^)^; long-chain *n*-3 PUFA^(^ ^14^ ^)^ and the Harris, von Schacky (HS)-*n*-3 index^(^ ^15^ ^)^.

**Fig. 1:**
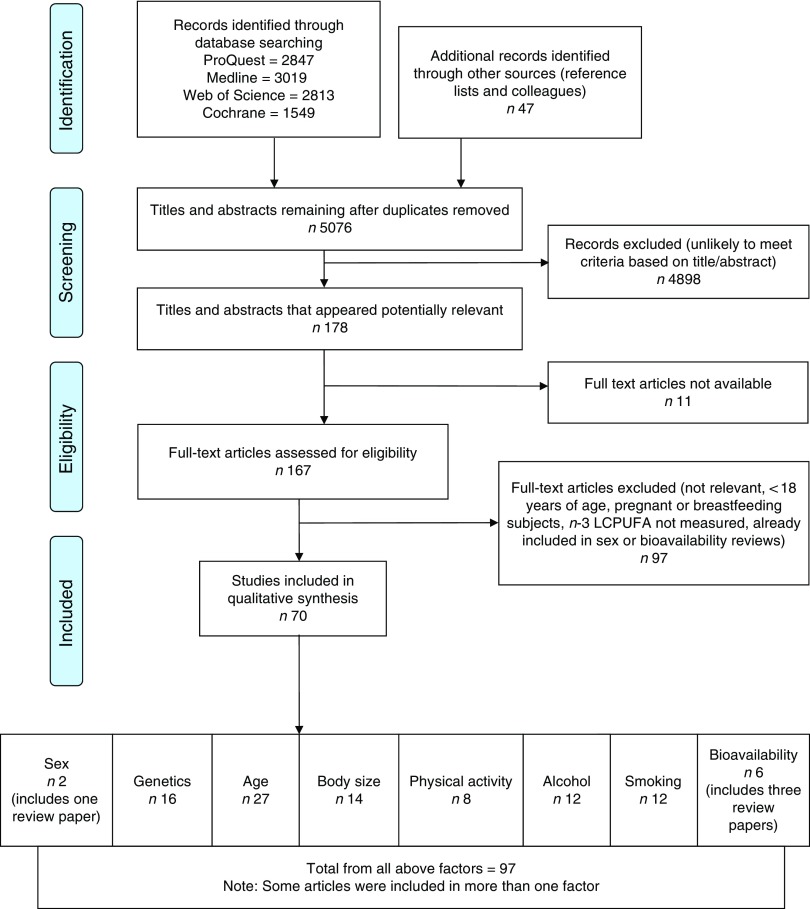
Flow diagram of systematic literature review search. The flow diagram outlines the identification, screening, eligibility and inclusion process of the systematic literature search. *n*-3 LCPUFA, *n*-3 long-chain PUFA.

For comparison between different studies, we used the available *n*-3 LCPUFA data and re-calculated them into erythrocyte EPA and DHA levels using the equations developed by Stark *et al.* ^(^ ^16^ ^)^ where applicable.

### Inclusion and exclusion criteria

The search results were screened based on the titles and abstracts. Titles and abstracts which suggested the study identified one or more factors that are associated with the *n*-3 LCPUFA levels were selected and screened for eligibility. Research studies met the inclusion criteria if (1) they were written in the English language, (2) they were conducted in humans, (3) the participants were at least 18 years of age, (4) the participants were not pregnant, (5) the *n*-3 LCPUFA levels were reported (EPA or DHA or both) and (6) they were cross-sectional studies or were intervention studies that included baseline data; the results from the effects of *n*-3 LCPUFA intervention studies were excluded (except for the factor ‘bioavailability’, because intervention studies are the only way to determine this). In addition, (7) relevant previous review publications were included if they focused on factors associated with/influencing the *n*-3 LCPUFA levels. In that case, only additional publications published after the release date of the review publications on this respective factor were included. Publications that did not meet these criteria based on abstract review were excluded, and those that did were read in detail to confirm their inclusion. Further studies were then obtained through hand searching the reference lists of these articles and applying the above eligibility criteria. Quality checks were performed and consensus on scores agreed on by all authors, using either the National Heart Lung and Blood Institute ‘Quality Assessment Tool for Observational Cohort and Cross-Sectional Studies’ (at https://www.nhlbi.nih.gov/health-topics/study-quality-assessment-tools)^(^ ^17^ ^)^ or the Effective Public Health Practice Project ‘Quality Assessment Tool For Quantitative Studies’ (at https://merst.ca/wp-content/uploads/2018/02/quality-assessment-tool_2010.pdf)^(^ ^18^ ^)^, depending on the type of study.

### Review of the literature

Eligible articles were categorised into groups, according to the factors that they covered. The groups were unmodifiable factors – ‘sex’, ‘genetics’ and ‘age’; modifiable factors – ‘body size’, ‘physical activity’, ‘alcohol’ and ‘smoking’; and bioavailability factors – ‘chemically bound form of supplement’, ‘krill oil *v.* fish oil’ and ‘conversion of plant-derived ALA to *n*-3 LCPUFA’. Some articles covered more than one factor and were therefore included in each group that they represented.

Four review articles were identified, wherein one evaluated the association of sex with *n*-3 LCPUFA levels^(^ ^19^ ^)^ and three reviewed the bioavailability factors^(^ ^20^ ^–^ ^22^ ^)^. We therefore did not execute a full systematic review of the factors sex and bioavailability.

## Results

The search returned 10 275 articles and after removal of duplicates 5076 articles remained. The flow diagram (Fig. 1) outlines the number of articles included after the screening and eligibility criteria were applied.

### Sex

A previous systematic literature review^(^ ^19^ ^)^ demonstrated differences in plasma DHA (expressed as weight/weight percentage of total plasma fatty acids) between sexes; namely, women had 0·12 % of total plasma fatty acids and 0·20 % of plasma phospholipids (PL) higher than men (*P*=0·002 and *P*<0·00001, respectively)^(^ ^19^ ^)^. In participants aged 13–50 years, the DHA values were significantly higher in women (0·16 % of total plasma fatty acids) compared with men; whereas the DHA values did not differ when aged over 50 years^(^ ^19^ ^)^. In high fish intake groups, sex differences in DHA did not exist; however, in low fish intake groups, the DHA was significantly higher in women (0·24 % of total plasma fatty acids)^(^ ^19^ ^)^.

Since the publication of the systematic literature review^(^ ^19^ ^)^, one large study^(^ ^23^ ^)^ showed that women from teens to aged 40 years had lower erythrocyte EPA and DPA compared with men, but women from teens to age 30 years had higher erythrocyte DHA levels compared with men.

### Heritability

One study^(^ ^10^ ^)^ identified that heritability (meaning the fraction of phenotype variability that can be attributed to genetic variation) explains 24 % of the variance of the *n*-3 LCPUFA levels, see online Supplementary Table S2.

### Genetics

Our systematic literature search revealed sixteen papers on the association of the factor ‘genetics’ and *n*-3 LCPUFA levels, as described subsequently.

#### Fatty acid desaturase

Nine studies were found, which looked at the relationship between fatty acid desaturase (FADS) genotypes and *n*-3 LCPUFA levels^(^ ^24^ ^–^ ^32^ ^)^. A minor allele carrier of a FADS SNP was negatively associated with plasma EPA in six studies^(^ ^24^ ^–^ ^26^ ^,^ ^29^ ^,^ ^31^ ^,^ ^32^ ^)^ and a negative association with DHA in three of those studies^(^ ^24^ ^,^ ^25^ ^,^ ^32^ ^)^. Three studies^(^ ^27^ ^,^ ^28^ ^,^ ^30^ ^)^ found no association between FADS minor allele carriers and plasma EPA or DHA. In essence, minor allele carriers for FADS1 and FADS2 resulted in decreased plasma levels of *γ*-linolenic acid (GLA, 18 : 3*n*-6), arachidonic acid (AA, 20 : 4*n*-6) and 20 : 5*n*-3 (EPA) (Table 1). The comparison of the major allele to the minor allele (homozygous or heterozygous plus homozygous) for FADS1 and FADS2 and their effects on plasma fatty acid levels are shown in Table 1.

**Table 1:** Comparison of major allele with minor allele (homozygous or heterozygous plus homozygous) for fatty acid desaturase (FADS)1 and FADS2 on fatty acid levels (Percentage increase or percentage decrease)*†

	rs174561 (FADS1)		rs174537 (C10orf10, FADS1)				rs174583 (FADS2)		rs3834458 (FADS2)	
Fatty acid	Gillingham^(^ ^26^ ^)^	Al-Hilal^(^ ^24^ ^)^	Tanaka (InCHIANTI)^(^ ^31^ ^)^	Tanaka (GOLDN)^(^ ^31^ ^)^	Al-Hilal^(^ ^24^ ^)^	FADS1 (average)	Gillingham^(^ ^26^ ^)^	Baylin^(^ ^32^ ^)^	Al-Hilal^(^ ^24^ ^)^	FADS2 (average)
18 : 2*n*-6	NS	↑ 3	↑ 7	↑ 13	NS	↑ 5	NS	NS	NS	NS
18 : 3*n*-6	↓ 61	↓ 19	NR	NR	↓ 19	↓ 33	↓ 62	↓ 53	↓ 20	↓ 45
20 : 2*n*-6	NR	NR	↓ 25	NR	NR	↓ 25‡	NR	↑ 19	NR	↑ 19‡
20 : 3*n*-6	NS	↑ 11	NR	NR	↑ 11	↑ 7	NS	NR	↑ 10	↑ 5
20 : 4*n*-6	↓ 37	↓ 18	↓ 27	↓ 12	↓ 18	↓ 22	↓ 37	↓ 39	↓ 18	↓ 31
18 : 3*n*-3	NS	↑ 8	↑ 10	↑ 14	NS	↑ 6	NS	NS	↑ 10	↑ 3
20 : 3*n*-3	NR	NR	NR	NR	NR	NR	NR	↑ 90	NR	↑ 90‡
20 : 5*n*-3	↓ 50	↓ 17	↓ 22	↓ 14	↓ 16	↓ 24	↓ 52	↓ 34	↓ 13	↓ 33
22 : 5*n*-3	NS	↓ 9	NR	↓ 7	↓ 10	↓ 7	NS	NR	↓ 9	↓ 5
22 : 6*n*-3	NS	↓ 11	NS	NS	↓ 10	↓ 4	NS	↓ 15	↓ 10	↓ 8

InCHIANTI, a study involving people in Chianti in Italy; GOLDN, Genetics of Lipid Lowering Drugs and Diet Network Study in the USA; NR, not reported.

* NS, for the calculation of the average, NS was taken as being 0.

† The data in Table 1 are the fatty acid data taken from the publications that reported fatty acid data and then expressed as percentage increase or percentage decrease in the fatty acid compared with the major allele (no mutation). This is a rough estimate of the magnitude of effect and therefore needs to be interpreted with caution.

‡ Limited data available as only one study reported this result.

#### Elongation of very-long-chain fatty acid 2

Three studies were identified that looked at the relationship between elongation of very-long-chain fatty acid (ELOVL)2 and EPA, DPA and DHA plasma levels^(^ ^26^ ^,^ ^31^ ^,^ ^33^ ^)^. Two studies^(^ ^31^ ^,^ ^33^ ^)^ observed lower plasma DHA levels in minor allele carriers, whereas one of them^(^ ^31^ ^)^ saw higher EPA levels in minor allele carriers. Another study^(^ ^26^ ^)^ found no association of ELOVL2 rs953413 and plasma fatty acids (Table 2). The comparison of the major allele to the minor allele (homozygous or heterozygous plus homozygous) for ELOVL2 and their effects on plasma *n*-3 LCPUFA are shown in Table 2.

**Table 2:** Comparison of major allele with minor allele (homozygous or heterozygous plus homozygous) for ELOVL2 on fatty acid levels (Percentage increase or percentage decrease)*†

	rs953413 (ELOVL2)				rs3734398 (ELOVL2)	rs2236212 (ELOVL2)	
Fatty acid	Gillingham^(^ ^26^ ^)^	Alsaleh^(^ ^33^ ^)^	Tanaka-InCHIANTI^(^ ^31^ ^)^	Tanaka-GOLDN^(^ ^31^ ^)^	Alsaleh^(^ ^33^ ^)^	Alsaleh^(^ ^33^ ^)^	ELOVL2 average
20 : 5*n*-3	NS	NS	↑ 14	NS	NS	NS	↑ 2
22 : 5*n*-3	NS	NS	NR	↓ 1·1	NS	NS	↓ 1
22 : 6*n*-3	NS	↓ 7	↓ 8	↓ 7	↓ 6	↓ 6	↓ 6

ELOVL2, elongation of very-long-chain fatty acid 2; InCHIANTI, a study involving people in Chianti in Italy; GOLDN, Genetics of Lipid Lowering Drugs and Diet Network Study in the USA; NR, not reported.

* NS, for the calculation of the average, NS was taken as being 0.

† The data in Table 2 are the fatty acid data taken from the publications that reported fatty acid data and then expressed as percentage increase or percentage decrease in the fatty acid compared with the major allele (no mutation). This is a rough estimate of the magnitude of effect and therefore needs to be interpreted with caution.

#### ApoE4

Only five studies were available on ApoE4, as shown in Supplementary Table S2, and the results did not suggest a strong relationship between ApoE4 and EPA or DHA plasma levels^(^ ^34^ ^–^ ^38^ ^)^. One study^(^ ^34^ ^)^ found ApoE4 carriers had higher plasma TAG EPA and DHA than non-carriers at baseline, but baseline EPA and DHA levels were not shown to be different in any of the participant groups in the additional four papers reviewed^(^ ^35^ ^–^ ^38^ ^)^ (online Supplementary Table S2).

### Age

**Fig. 2:**
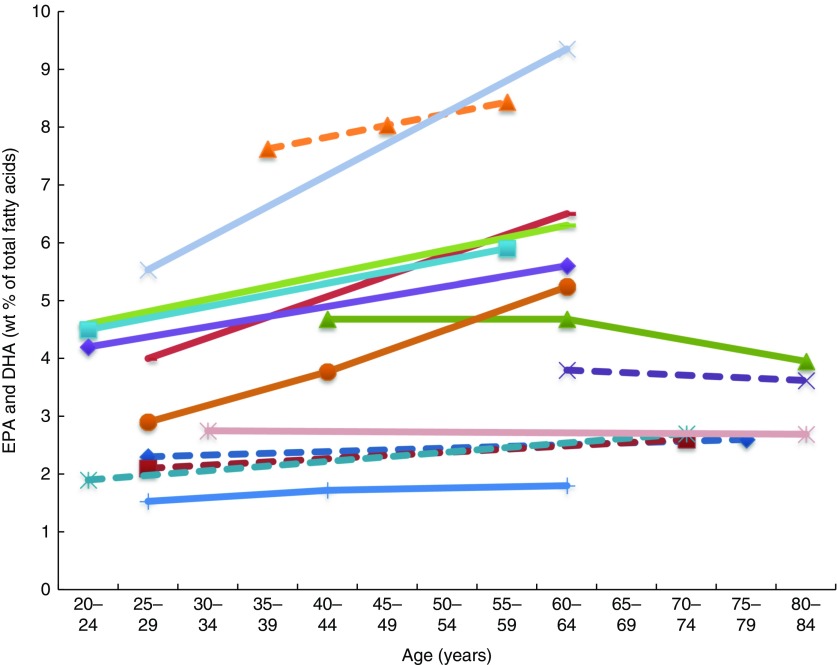
Plasma EPA levels and DHA (wt% of total fatty acids) of different age groups from studies reviewed in this systematic literature review. Each line represents a different study (population) and each symbol on a line represents an age group. Full line = significant difference between age groups measured in that study. Broken line = no significant difference between age groups measured in that study. 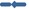, Plourde *et al*.^(^ ^43^ ^)^ NS; 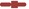, Vandal *et al*.^(^ ^42^ ^)^ NS; 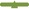, Sfar *et al*. (women)^(^ ^54^ ^)^; 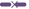, Sfar *et al*. (men)^(^ ^54^ ^)^ NS; 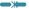, Fortier *et al*.^(^ ^40^ ^)^ NS; 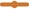, Dewailly *et al*.^(^ ^58^ ^)^; 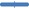, Dewailly *et al*.^(^ ^59^ ^)^; 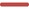, Rees *et al*. g1^(^ ^41^ ^)^; 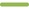, Rees *et al*. g2^(^ ^41^ ^)^; 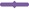, Rees *et al*. g3^(^ ^41^ ^)^; 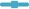, Rees *et al*. g4^(^ ^41^ ^)^; 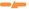, Kuriki *et al*.^(^ ^46^ ^)^ NS; 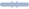, Dewailly *et al*.^(^ ^61^ ^)^; 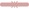, Babin *et al*.^(^ ^57^ ^)^ NS.

**Fig. 3:**
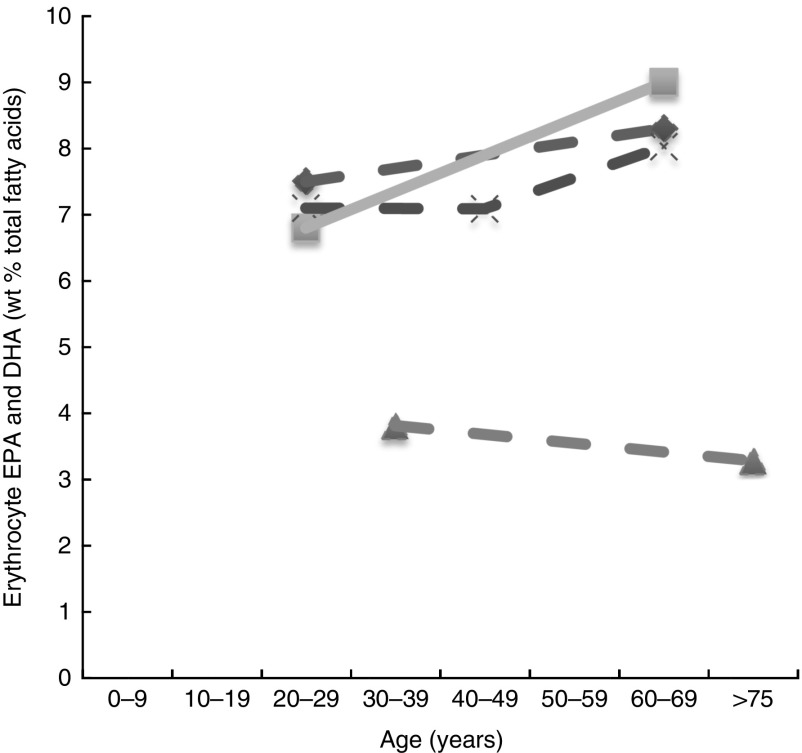
Erythrocyte EPA levels and DHA (wt% of total fatty acids) of different age groups from studies reviewed in this systematic literature review. Each line represents a different study (population) and each symbol on a line represents an age group. Full line=significant difference between age groups measured in that study. Broken line=no significant difference between age groups measured in that study. 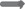, Kawabata *et al.* ^(^ ^53^ ^)^ (women subjects) NS; 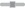, Kawabata *et al.* ^(^ ^53^ ^)^ (male subjects); 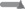, Babin *et al.* ^(^ ^57^ ^)^ NS; 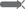, Walker *et al.* ^(^ ^39^ ^)^ NS.

Twenty-six articles, which looked at ‘age’ as a factor associated with *n*-3 LCPUFA levels, were included^(^ ^10^ ^,^ ^23^ ^,^ ^39^ ^–^ ^62^ ^)^. Most publications reported plasma EPA and DHA and only four studies reported erythrocyte levels of EPA and DHA^(^ ^23^ ^,^ ^39^ ^,^ ^53^ ^,^ ^57^ ^)^. Twenty-four found a positive association, one found no association^(^ ^57^ ^)^, and another^(^ ^54^ ^)^ an inverse association between age 40–60 and age 61–82 years and *n*-3 LCPUFA (specifically DHA levels in elderly women) (Figs. 2 and 3). See online Supplementary Table S3 for detailed information on the range of age groups and the relevant outcomes for each of the twenty-six studies reviewed.

Plasma EPA and DHA levels are positively associated with age in the majority of studies. Erythrocyte EPA and DHA levels only tended to be positively associated with age in adults^(^ ^39^ ^,^ ^53^ ^)^, with only statistical significance shown in men^(^ ^53^ ^)^. One study did not show associations with increased age^(^ ^57^ ^)^. One large study^(^ ^23^ ^)^ showed that the net effect on erythrocyte EPA and DHA was an overall 7 % increase per decade up to 70 years of age and not much change after that.

### Body size

Of the fourteen studies that looked for associations between the factor ‘body size’ and *n*-3 LCPUFA levels^(^ ^10^ ^,^ ^11^ ^,^ ^47^ ^,^ ^49^ ^,^ ^52^ ^,^ ^58^ ^–^ ^61^ ^,^ ^63^ ^–^ ^67^ ^)^, eight studies used ‘BMI’^(^ ^11^ ^,^ ^47^ ^,^ ^49^ ^,^ ^52^ ^,^ ^60^ ^,^ ^63^ ^–^ ^65^ ^)^, three used ‘girth’^(^ ^58^ ^,^ ^59^ ^,^ ^61^ ^)^ and three studies^(^ ^10^ ^,^ ^66^ ^,^ ^67^ ^)^ used both to compare the weight-based association and *n*-3 LCPUFA. Despite the strong correlations, we chose to report BMI and girth in relation to *n*-3 LCPUFA separately, because they provide different information about the participants’ fat distribution and require very different methodology for measurement.

#### BMI

Overall, of the eleven cross-sectional studies that investigated the association of BMI and *n*-3 LCPUFA levels, five identified negative associations^(^ ^10^ ^,^ ^49^ ^,^ ^64^ ^,^ ^66^ ^,^ ^67^ ^)^, whereas six found no association^(^ ^11^ ^,^ ^47^ ^,^ ^52^ ^,^ ^60^ ^,^ ^63^ ^,^ ^65^ ^)^.

As shown in Table 3, it appears that there is no association when erythrocyte EPA and DHA is >7 % of total fatty acids and that there is a negative association when it is lower than 5·6 % of the total fatty acids (Table 3). An exception to this is the study by Block *et al.* ^(^ ^52^ ^)^ in which the mean erythrocyte EPA and DHA was 4·3 % and no association was found, wherein this population group comprised mostly overweight and obese (Table 3).

**Table 3:** Overview of studies investigating associations between BMI and *n*-3 long-chain PUFA levels presented in order of decreasing erythrocyte EPA and DHA

Reference	Subjects (*n*)	BMI range and (mean)	Erythrocyte EPA and DHA*: range and (mean)	Association
Ogura^(^ ^47^ ^)^	75	16·5–37·8 kg/m^2^ (26·2 kg/m^2^)	Range not given (9·6 %)	No
Makhoul^(^ ^65^ ^)^	330	Range not given (28·1 kg/m^2^) 71 % overweight or obese	Erythrocyte EPA range: 0·2–9·6 % Erythrocyte DHA range: 1·6–10·3 % EPA+DHA range not given (9·6 %)	No
Itomura^(^ ^60^ ^)^	456	16–38 kg/m^2^ (22·5 kg/m^2^)	3·9–12·9 % (8·5 %)	No
Sala-Vila^(^ ^11^ ^)^	198	21·2–38·5 kg/m^2^ (29·2 kg/m^2^)	Interquartile range: 6·1–8·1 % (7·1 %)	No
Kuriki^(^ ^63^ ^)^	106	Range not given (female mean 21·5 kg/m^2^) (male mean 22·3 kg/m^2^)	Range not given (7·1 %†)	No
Harris^(^ ^10^ ^)^	3196	Range not given (28·4 kg/m^2^)	Range not given (5·6 %)	Negative
Howe^(^ ^67^ ^)^	476	18–59 kg/m^2^ (female mean 34 kg/m^2^) (male mean 31·4 kg/m^2^)	Range not given (female 5·3 %) (male 5·1 %)	Negative
Sands^(^ ^49^ ^)^	163	18–47 kg/m^2^ (26·2 kg/m^2^)	1·7–12·4 % (4·9 %)	Negative
Block^(^ ^52^ ^)^	704	Range not given (28 kg/m^2^) Study reports most participants were overweight or obese	Range not given (4·3 %)	No
Cazzola^(^ ^64^ ^)^	100	Range and mean not given	4·2–6·3 % (3·7 %)	Negative
Micallef^(^ ^66^ ^)^	124	20–40 kg/m^2^ (mean not given) Twenty-one subjects between 20 and 24·9 kg/m^2^ Forty subjects between 25 and 29·9 kg/m^2^ Sixty-three subjects between 30 and 40 kg/m^2^	Range and mean not given	Negative

* Plasma levels were converted to erythrocyte EPA+DHA using the Stark *et al.* ^(^ ^16^ ^)^ equation.

#### Girth

Six studies were identified dealing with girth and *n*-3 LCPUFA levels. Three studies^(^ ^58^ ^,^ ^59^ ^,^ ^61^ ^)^ showed positive associations. Three studies^(^ ^10^ ^,^ ^66^ ^,^ ^67^ ^)^ found inverse associations; one of them^(^ ^66^ ^)^ found this only in the obese group, another study^(^ ^67^ ^)^ found this only in females, whereas the third study^(^ ^10^ ^)^ found that 1sd increase (14·7 cm) in girth was associated with 2 % lower *n*-3 LCPUFA status.

Given the small number of studies available for review and the differing results between studies, the relationship between girth and *n*-3 LCPUFA levels remains inconclusive.

### Physical activity

Many studies observed associations between exercise and *n*-3 LCPUFA levels; however, not all studies included EPA and DHA in their analyses^(^ ^68^ ^)^. Therefore, only eight studies were included^(^ ^11^ ^,^ ^60^ ^,^ ^63^ ^,^ ^69^ ^–^ ^73^ ^)^ in this review. Studies that investigated the effect of acute exercise were excluded.

One cross-sectional study that compared muscle fatty acids in male endurance athletes (mean training time of 74 (SD 24) min/d) with sedentary men (no regular physical activity) showed that DHA was approximately 30 % higher in male endurance athletes^(^ ^70^ ^)^.

Two studies found a positive association^(^ ^11^ ^,^ ^71^ ^)^, two studies found a negative association^(^ ^60^ ^,^ ^69^ ^)^, whilst four studies^(^ ^63^ ^,^ ^70^ ^,^ ^72^ ^,^ ^73^ ^)^ found no association between *n*-3 LCPUFA levels and physical activity (Table 4).

**Table 4:** Cross-sectional studies looking at differences in *n*-3 long-chain PUFA levels at different physical activity levels

Reference	Sex	Subjects (*n*)	Exercise	Biomarkers reported	Findings
Sala-Vila^(^ ^11^ ^)^	Women and men	198	Not specified	Whole blood	Physical activity positively ↑ associated with EPA and DHA
Kamada^(^ ^71^ ^)^	Men	6 long-distance runners 9 sprinters 10 sedentary controls	Athletes *v.* controls	Erythrocytes	EPA higher ↑ in long-distance runners compared with controls No difference in DHA levels between groups
Itomura^(^ ^60^ ^)^	Men and women	456	Not specified	Erythrocytes	Physical activity negatively ↓ associated with EPA and DHA
Sumikawa^(^ ^69^ ^)^	Sex not specified	12 rowers 9 sedentary controls	Athletes *v.* controls	Erythrocyte PC PS	Athletes had lower ↓ DHA composition in PS No difference in PC DHA levels EPA not measured
Andersson^(^ ^70^ ^)^	Men	15 endurance athletes 16 sedentary controls	Endurance athletes *v*. untrained	Serum	No differences in EPA or DHA between groups
Arsic^(^ ^72^ ^)^	Women	15 waterpolo players 19 footballers 14 sedentary controls	Athletes *v.* controls	Erythrocytes and plasma	No differences in EPA or DHA between groups
Kuriki^(^ ^63^ ^)^	Women and men	84	Not specified	Plasma	Physical activity was not associated with EPA and DHA
Tepsic^(^ ^73^ ^)^	Men	23 basketballers 24 footballers 16 sedentary controls	Athletes *v.* sedentary controls	Erythrocytes and plasma	No differences in EPA or DHA between groups

PC, phosphatidylcholine; PS, phosphatidylserine.

### Alcohol intake

Twelve papers were suitable for inclusion when looking at the association between alcohol and *n*-3 LCPUFA levels^(^ ^11^ ^,^ ^58^ ^,^ ^59^ ^,^ ^61^ ^,^ ^63^ ^,^ ^74^ ^–^ ^80^ ^)^. Six papers identified positive associations^(^ ^59^ ^,^ ^74^ ^–^ ^76^ ^,^ ^78^ ^,^ ^79^ ^)^; three found no association^(^ ^11^ ^,^ ^63^ ^,^ ^77^ ^)^ and four studies found negative associations^(^ ^58^ ^,^ ^61^ ^,^ ^76^ ^,^ ^80^ ^)^. One study^(^ ^76^ ^)^ is mentioned twice as they found erythrocyte PL EPA to be positively associated, whereas DHA was negatively associated.

#### Wine and *n*-3 long-chain PUFA levels

**Fig. 4:**
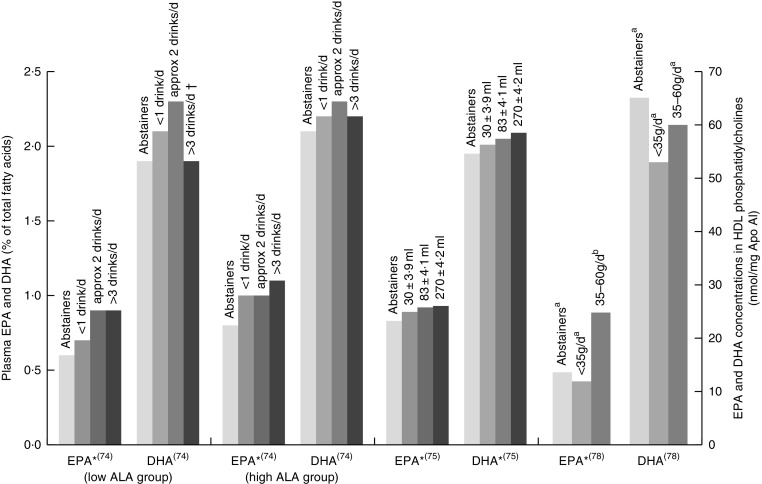
EPA and DHA levels in alcohol abstainers *v.* wine drinkers. Bar graph presents plasma EPA and DHA (percentage of total fatty acids) for de Lorgeril^(^ ^74^ ^)^ and di Giuseppe^(^ ^75^ ^)^ studies and EPA and DHA concentration in HDL phosphatidylcholines for the Perret study^(^ ^78^ ^)^. *P<0·05. †DHA intake was approximately 3× lower in subjects who drank >3 drinks per d compared with other subjects. ^a,b^Bars in the same study with unlike letters have significantly different fatty acid levels. ALA, *α*-linolenic acid.

Six papers found positive associations with wine and *n*-3 LCPUFA levels, and there were no studies showing negative or no associations. Three studies had cohorts that of mostly (>88 %) wine drinkers^(^ ^74^ ^,^ ^78^ ^,^ ^79^ ^)^, one study separated wine from beer or spirits and found that drinking ‘only’ wine was positively associated with *n*-3 LCPUFA levels but drinking ‘only’ beer or spirits was not^(^ ^75^ ^)^; and two studies^(^ ^59^ ^,^ ^76^ ^)^ did not mention the type of alcohol consumed but consisted of participants living in locations (France and Quebec) where wine is the most regularly consumed alcoholic beverage^(^ ^74^ ^,^ ^81^ ^)^. Fig. 4 shows the increases in plasma EPA or DHA associated with increasing wine intake among three studies that had sufficient data^(^ ^74^ ^,^ ^75^ ^,^ ^78^ ^)^. The optimal amount of wine consumption seems to plateau between two to three glasses per d (Fig. 4). In addition, it was found that erythrocyte PL EPA was positively associated (*β*=0·182, *P*=0·011) with alcohol intake in a French cohort, whereas DHA was negatively associated (*β*=–0·218, *P*=0·01)^(^ ^76^ ^)^ and one study^(^ ^79^ ^)^ found significantly higher DHA in phosphatidylethanolamine among wine drinkers compared to non-drinkers (*P*<0·05) but no differences in EPA. Whilst five studies found increases in EPA with increasing alcohol (mostly wine) intake^(^ ^59^ ^,^ ^74^ ^,^ ^75^ ^,^ ^78^ ^,^ ^79^ ^)^, Simonetti *et al.* ^(^ ^79^ ^)^ and Di Giuseppe *et al*.^(^ ^75^ ^)^ were the only studies to find a positive association between wine intake and plasma DHA.

#### Alcohol type not further specified

**Fig. 5:**
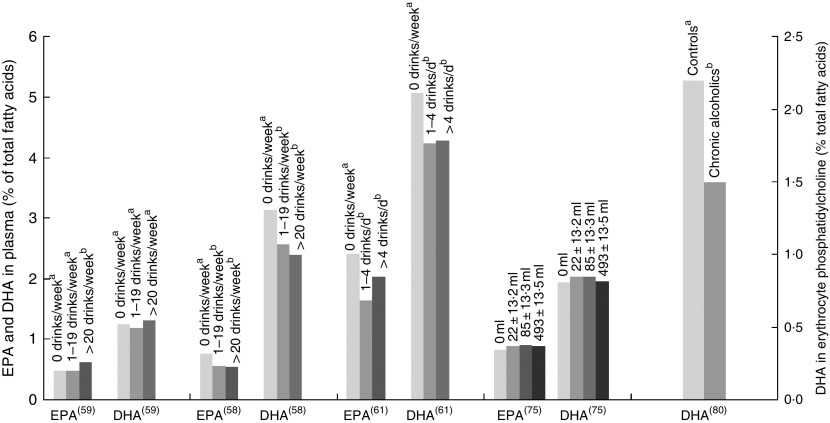
EPA and DHA levels at different alcohol intake. di Giuseppe^(^ ^75^ ^)^ shows EPA and DHA levels for beer and spirit drinkers. Alcohol type was not reported for the four other studies shown in the bar graph. Bar graph presents plasma EPA and DHA (percentage of total fatty acids) for Dewailly^(^ ^58^ ^,^ ^59^ ^,^ ^61^ ^)^ and di Giuseppe^(^ ^75^ ^)^ studies and DHA concentration in HDL phosphatidylcholines for the Alling study^(^ ^80^ ^)^. ^a,b^ Bars in the same study with unlike letters are significantly different from each other.

Two studies^(^ ^11^ ^,^ ^77^ ^)^ found no association between alcohol and *n*-3 LCPUFA levels, whereas one study^(^ ^63^ ^)^ identified a positive association in females only. Three studies found negative associations with alcohol intake and EPA and/or DHA levels (Fig. 5)^(^ ^58^ ^,^ ^61^ ^,^ ^80^ ^)^. None of these studies reported the type of alcohol consumed by participants, and relevant intake surveys or research to indicate the types of drinks most commonly consumed by these populations have not been reported.

### Smoking

Twelve studies were identified^(^ ^10^ ^,^ ^11^ ^,^ ^49^ ^,^ ^52^ ^,^ ^58^ ^–^ ^61^ ^,^ ^63^ ^,^ ^77^ ^,^ ^82^ ^,^ ^83^ ^)^ of which eight found a negative association between smoking and *n*-3 LCPUFA levels^(^ ^10^ ^,^ ^11^ ^,^ ^52^ ^,^ ^58^ ^,^ ^59^ ^,^ ^77^ ^,^ ^82^ ^,^ ^83^ ^)^ and four studies found no association^(^ ^49^ ^,^ ^60^ ^,^ ^61^ ^,^ ^63^ ^)^.

Of the twelve studies, four studies^(^ ^58^ ^,^ ^59^ ^,^ ^61^ ^,^ ^83^ ^)^ provided numerical data on FA levels of smokers and non-smokers. Using the Stark *et al.* ^(^ ^16^ ^)^ equation or the already available data^(^ ^10^ ^,^ ^52^ ^,^ ^83^ ^)^, erythrocyte EPA and DHA levels in smokers and non-smokers were determined and ranged from 6 to 17 % lower in smokers compared with non-smokers. Three studies had no numerical data^(^ ^11^ ^,^ ^77^ ^,^ ^82^ ^)^, but each also reported lower *n*-3 LCPUFA levels among smokers compared with non-smokers.

### Bioavailability factors

#### Chemically bound form of *n*-3 supplement

One study^(^ ^22^ ^)^ reviewed the different factors associated with the bioavailability of *n*-3 LCPUFA. This review and another subsequently published study^(^ ^84^ ^)^ showed that the chemically bound form TAG are more bioavailable than ethyl ester (EE) forms and that there is no enough evidence to suggest that PL (like krill oil) are more bioavailable than the TAG form from fish oil. They also showed that matrix effects such as sufficient amounts of fat in the meal have the greatest bioavailability effect of up to three times higher^(^ ^85^ ^)^. It appears that some studies from the Schuchardt review^(^ ^22^ ^)^ showed that the galenic form (i.e. microencapsulation, emulsification) had an effect on increased bioavailability (e.g. up to 4-fold^(^ ^86^ ^)^) of emulsification and microencapsulation compared with oil, whilst others showed no effect.

#### Krill oil *v.* fish oil

A review^(^ ^20^ ^)^ identified fourteen articles comparing krill oil and fish oil, and they found that some studies showed increased bioavailability with krill oil *v.* fish oil, but other studies did not show any difference and concluded that more studies are needed. Following the publishing of this review, two more clinical trials have been published. One study^(^ ^87^ ^)^ found no difference in bioavailability of fish oil (TAG-rich or EE-rich) and krill oil supplements when identical doses were used in a 4-week intervention. Another study examined the amount of PL in krill oil^(^ ^88^ ^)^ and showed no difference between krill oil with high PL content and krill oil with low PL content in plasma *n*-3 LCPUFA levels. However, the high PL supplement significantly increased erythrocyte EPA, EPA+DHA and *n*-3 PUFA concentrations compared with the low PL supplement^(^ ^88^ ^)^.

#### Conversion of plant-derived *n*-3, *α*-linolenic acid to *n*-3 long-chain PUFA

The International Society for the Study of Fatty Acids and Lipids (ISSFAL) statement five (http://www.issfal.org/statement-5) concludes that ‘With no other changes in diet, improvement of blood DHA status can be achieved with dietary supplements of preformed DHA, but not with supplementation of ALA, EPA, or other precursors’. Furthermore, a comprehensive review on the metabolism of ALA and stearidonic acid (SDA, 18 : 4*n*-3)^(^ ^21^ ^)^ suggests that each 1 g increase in ALA intake results in approximately 10 % relative increase in EPA plasma PL content, whereas no change occurs in plasma PL DHA content. With high intake of EPA and DHA, however, the metabolism of ALA to EPA and DHA appears to become down-regulated^(^ ^21^ ^)^. High intakes of linoleic acid (LA, 18 : 2*n*-6) can also impact the metabolism of ALA to its longer chain metabolites. Furthermore, increased LA intake have been shown to decrease the metabolism of ALA to EPA^(^ ^21^ ^)^.

It was also demonstrated^(^ ^21^ ^)^ that SDA intake between 0·25 and 2 g/d can increase plasma EPA anywhere from 19 to 190 %. No superior ability was noted for SDA to increase the DHA levels, and some studies actually noted a decrease in DHA levels when participants consumed SDA^(^ ^21^ ^)^.

## Discussion

Besides dietary intake, many factors affect *n*-3 LCPUFA levels. Generally women have higher plasma DHA compared with men^(^ ^19^ ^,^ ^23^ ^)^, and this appears to be independent of diet^(^ ^89^ ^)^. Women also have increased levels of EPA derived from ALA^(^ ^90^ ^)^ which is believed to be indicative of increased synthesis^(^ ^91^ ^,^ ^92^ ^)^. The sex differences can be explained by (1) decreased rates of ALA *β*-oxidation^(^ ^91^ ^,^ ^92^ ^)^, therefore making more ALA available for metabolism to DHA; (2) women having more DHA in their adipose tissue^(^ ^62^ ^)^ and therefore can mobilise more DHA (but it is still not known whether this occurs in non-pregnant women); (3) the fasting state wherein NEFA are released from adipose tissue; and women have increased NEFA compared with men^(^ ^89^ ^)^, and this is likely due to increased adipose tissue stores; (4) the total fractional excursions of EPA, DPA and DHA in plasma phosphatidylcholine were greater in younger women (74 %) compared with men (59·6 %)^(^ ^93^ ^)^ and (5) the influence of different sex hormones on the *n*-3 pathway^(^ ^94^ ^)^, which is likely due to the up-regulation mechanism of oestrogen on the desaturase–elongase *n*-3 pathway and a possible down-regulation by testosterone^(^ ^94^ ^)^. This may partially explain why women >50 years of age have DHA levels that are comparable with men. Increased requirements during pregnancy and lactation could provide a biological explanation to why higher DHA levels have been observed in women^(^ ^21^ ^)^. Certainly, in very early pregnancy, the requirement to increase maternal circulating DHA at the time of the neural tube closure is likely due to increased synthesis of DHA from ALA as well as an increase in the mobilisation of DHA from maternal adipose and other tissues^(^ ^3^ ^)^. Later pregnancy shows maternal erythrocyte DHA levels being 38 % higher in the third trimester (3·85 %) compared with the post-partum levels (2·79 %)^(^ ^95^ ^)^, demonstrating that the magnitude of effect of increased DHA in pregnancy is much higher than the differences seen in non-pregnant women *v.* men.

**Fig. 6:**
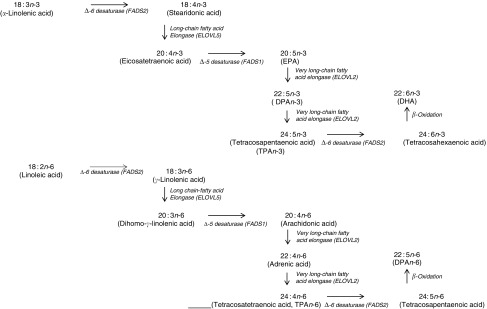
Mammalian PUFA synthesis pathway showing the *n*-3 PUFA pathway and the *n*-6 PUFA pathway, including the enzymes responsible for the elongation and desaturation steps. ELOVL, elongation of very long-chain fatty acid; DPA, docosapentaenoic acid.

In terms of genetics, when comparing the baseline cross-sectional *n*-3 LCPUFA levels between major and minor allele carriers for FADS1 and FADS2, we deduced that there was decreased enzyme activity at the first Δ-6 desaturase and Δ-5 desaturase in the minor allele carriers (Fig. 6) and therefore this resulted in decreased levels of EPA, GLA and AA. However, as the FADS1 and FADS2 genotypes are strongly associated, controlling for one FADS1 or FADS2 would be sufficient. Similarly, when comparing baseline cross-sectional *n*-3 LCPUFA levels between major and minor allele for ELOVL2, we deduced that there was decreased enzyme activity between EPA and DHA in the minor allele carriers (Fig. 6), which explains the reduced DHA levels seen with these mutations. These findings are supported by a meta-analysis^(^ ^96^ ^)^. Dietary supplementation with pre-formed EPA and DHA (1·8 g/d) may overcome these decreased enzyme activities as EPA and DHA in minor allele carriers were 26–30 and 8–9 % higher, respectively, than non-carriers^(^ ^33^ ^)^. Therefore, in supplementation trials with pre-formed EPA and DHA, it may not be necessary to measure the minor allele SNP. Furthermore, heritability explains 24 % of the variance of *n*-3 LCPUFA levels^(^ ^10^ ^)^, which is more relevant than genetics alone.

It appears that higher plasma EPA and DHA levels are associated with increased age, that is, up until 70 years of age^(^ ^10^ ^,^ ^23^ ^,^ ^39^ ^–^ ^41^ ^,^ ^44^ ^–^ ^53^ ^,^ ^55^ ^,^ ^56^ ^,^ ^58^ ^–^ ^62^ ^)^. Based on studies that reported no differences in DHA levels between elderly and young groups^(^ ^42^ ^,^ ^43^ ^)^, those studies that included elderly participants of over 70 years of age^(^ ^42^ ^,^ ^43^ ^,^ ^57^ ^)^ and women aged 40–82 years showed lower levels of plasma EPA and DHA in the 60- to 82-year-olds compared with the 40- to 60-year-olds^(^ ^54^ ^)^, which could be explained by the 60- to 82-year-old women being post-menopausal and therefore likely to have lower oestrogen levels and hence lower synthesis of DHA from ALA.

Negative associations between *n*-3 LCPUFA levels and BMI have been found in participants with erythrocyte EPA and DHA of 5·6 % or lower (Table 3). No associations have been found with higher than 7 % erythrocyte EPA and DHA and BMI. With the contradictory evidence in terms of girth and *n*-3 LCPUFA levels and taking into account that the majority of populations’ erythrocyte EPA and DHA is likely to be lower than 5·6 % of the total fatty acids, future studies should take BMI into account in their analyses.

The following different mechanisms have been suggested for the negative associations occasionally observed: (1) higher susceptibility to peroxidation in overweight and obese individuals, compared with normal-weight individuals^(^ ^64^ ^,^ ^97^ ^,^ ^98^ ^)^, (2) individuals with higher BMI may be more likely to consume lower intakes of *n*-3 LCPUFA^(^ ^63^ ^)^, although the opposite was found^(^ ^49^ ^)^, (3) alternatively, a relationship between weight and dose might exist for *n*-3 LCPUFA^(^ ^99^ ^)^, as supported by a study showing a three-unit rise in BMI is associated with a decrease in the *n*-3 LCPUFA status by 0·3 units, independent of fish intake^(^ ^49^ ^)^.

There is no conclusive evidence on whether an association exists between physical activity and *n*-3 LCPUFA, though several potential underlying mechanisms have been suggested^(^ ^72^ ^,^ ^73^ ^,^ ^100^ ^,^ ^101^ ^)^, and more research is warranted. Potential associations might depend on type, duration and intensity of physical activity, as higher DHA in skeletal muscle was observed in endurance athletes compared with sedentary controls^(^ ^70^ ^)^ but not in participants who followed a low-intensity exercise programme for 6 weeks^(^ ^102^ ^)^. Differences in fatty acid composition between athletes from different sports have also been found^(^ ^72^ ^,^ ^73^ ^)^.

The association between alcohol consumption and *n*-3 LCPUFA levels is either negative or neutral, except for wine consumption where there is a positive association^(^ ^59^ ^,^ ^74^ ^–^ ^76^ ^,^ ^78^ ^,^ ^79^ ^)^, in particular for EPA^(^ ^59^ ^,^ ^74^ ^–^ ^76^ ^,^ ^78^ ^)^. Studies that did not demonstrate the type of alcohol consumed showed conflicting results between papers^(^ ^11^ ^,^ ^58^ ^,^ ^61^ ^,^ ^63^ ^,^ ^77^ ^,^ ^80^ ^)^; the majority of these showed negative associations with alcohol intake^(^ ^58^ ^,^ ^61^ ^,^ ^76^ ^,^ ^80^ ^)^. Mechanisms for the negative associations between alcohol intake and *n*-3 LCPUFA are still not fully understood in humans; however, animal and *in vitro* studies propose lipid peroxidation and changes in desaturase activities^(^ ^103^ ^–^ ^105^ ^)^. Different findings observed between the studies might be due to the differences in amounts of alcohol consumed, the regularity with which alcohol is consumed and whether participants consumed more quantity of alcohol for prolonged periods^(^ ^106^ ^)^.

The positive associations between wine drinking and *n*-3 LCPUFA seem to partly contradict the mechanisms discussed above. This poses the question whether components in wine other than alcohol might be responsible for the positive associations observed. This warrants further research but could explain why one study^(^ ^75^ ^)^ saw no association for beer or spirits and *n*-3 LCPUFA but a positive association between wine and *n*-3 LCPUFA. Diet is the main contributor of *n*-3 LCPUFA levels^(^ ^10^ ^)^; and therefore, differences in dietary intake between drinkers and non-drinkers could also influence associations, though this is not uniformly supported by the literature^(^ ^11^ ^,^ ^61^ ^,^ ^63^ ^,^ ^74^ ^,^ ^107^ ^)^. Any research on *n*-3 LCPUFA levels should capture not only alcohol consumption but also the type and amount of alcohol.

Smoking is associated with a lower erythrocyte EPA and DHA (from 6 to 17 % lower) and thus smoking is a factor that needs to be controlled for in research studies. A plausible reason for lower erythrocyte EPA and DHA in smokers compared with non-smokers could be diet^(^ ^108^ ^–^ ^110^ ^)^, though others suggest the involvement of non-dietary factors, as they found this negative association regardless of the dietary intake^(^ ^10^ ^,^ ^52^ ^,^ ^77^ ^)^. It has been suggested that the pro-oxidative state caused by smoking degrades PUFA^(^ ^111^ ^)^; *n*-3 LCPUFA oxidation has been shown to be increased in smokers. Of the four studies that showed no association between smoking and *n*-3 LCPUFA, three consisted of cohorts with high *n*-3 LCPUFA intakes, providing mean annual daily intake of EPA and DHA of 1293 mg^(^ ^60^ ^)^, 2115 mg^(^ ^61^ ^)^ and 885 mg/d^(^ ^63^ ^)^, and one study had a very low number (*n* 13 of 163) of smokers in their cohort^(^ ^49^ ^)^. It could be that the high intake of *n*-3 LCPUFA negates the effect of smoking on erythrocyte EPA and DHA. It should be noted, however, that in the Spanish cohort^(^ ^11^ ^)^ the erythrocyte EPA and DHA were very similar to the cohort of Nunavik Inuit^(^ ^61^ ^)^, thereby showing a negative association between smoking and erythrocyte EPA and DHA.

The major contributor to increased bioavailability of *n*-3 LCPUFA appears to be fat in the meal when supplements are being taken. The biological plausibility is that the fat in the meal stimulates the release of pancreatic lipase necessary for fat digestion^(^ ^112^ ^)^. The increased bioavailability of the emulsified forms compared with the larger oil droplets supports that these emulsified forms are more readily available for pancreatic lipase. The limited evidence suggests that the EE form of *n*-3 LCPUFA is less bioavailable compared with the TAG form. Compared to low PL krill oil, high PL krill oil resulted in higher erythrocyte EPA and DHA^(^ ^88^ ^)^, which has been demonstrated in only one study. There is no definitive evidence to support that krill oil or fish oil is superior to the other in terms of bioavailability as studies to date (1) were underpowered^(^ ^22^ ^)^, (2) used different doses of EPA and DHA^(^ ^20^ ^)^ and (3) involved short duration of supplementation, that is, for 4 weeks^(^ ^87^ ^)^, which was not enough, given the mean erythrocyte lifespan of 115 d^(^ ^113^ ^)^. One cross-over study^(^ ^22^ ^)^ noted the high standard deviations, even though each person was their own control, thereby contribute to the lack of definitive evidence.

Supplementation with ALA increases plasma EPA but not DHA, and high intake of LA reduces the conversion of ALA to EPA^(^ ^21^ ^)^. Limited evidence suggests that SDA supplementation increases plasma EPA to a greater extent than supplementation with ALA, but SDA supplementation does not increase plasma DHA levels^(^ ^21^ ^)^. More research is warranted on SDA.

Given all the non-dietary factors that are associated with *n*-3 LCPUFA levels as discussed above and summarised in Table 5 below (but keep in mind that fish, seafood and *n*-3 supplement consumption have the biggest influence), it is difficult to stipulate how these non-dietary factors relate to each other, as these factors are associated with *n*-3 LCPUFA levels and do not show cause and effect. Furthermore, no scientific evidence is available that shows the relationship between these factors; however, one could speculate about these relationships. The global data of the prevalence of smoking and drinking alcohol are higher among males compared with females^(^ ^114^ ^–^ ^116^ ^)^. Therefore, future investigators should consider when studying males, who smoke more than women do and drink more alcohol than wine, that their *n*-3 LCPUFA levels would likely be lower than that in females who smoke less and drink wine rather than beer/spirits^(^ ^114^ ^–^ ^119^ ^)^. Furthermore, the prevalence of overweight and obesity is high in many western countries^(^ ^120^ ^,^ ^121^ ^)^; and taken together with the low *n*-3 LCPUFA levels globally^(^ ^122^ ^)^, the negative association between BMI and *n*-3 LCPUFA is also of concern. Overall, given the potential lower levels of *n*-3 LCPUFA in males, they are likely to see a benefit through *n*-3 LCPUFA supplementation. Conversely, given the *n*-3 LCPUFA levels may be higher in females compared with males, researchers need to be careful not to reach the potential ceiling effect. Given that *n*-3 LCPUFA levels are positively associated with age, researchers need to carefully consider the age range within studies. The best advice would be to measure the *n*-3 LCPUFA levels in all types of research including at baseline and post-supplementation in clinical trials.

**Table 5:** Summary of factors affecting *n*-3 long-chain PUFA (LCPUFA) levels

Factor	Conclusion 1 and quantification	Conclusion 2 and quantification	Conclusion 3 and quantification	Should take factor into account yes or no
Sex	DHA in all women is 0·12 % of total plasma fatty acids higher than men	DHA in women aged 13–50 years is 0·16 % of total plasma fatty acids higher than men	In low fish consumers, DHA in women is 0·24 % of total plasma fatty acids higher than men	Yes
Genetics	Mutations in FADS1 and FADS2 generally result in decreased levels of EPA, GLA and AA	Supplementation of EPA and DHA at doses of 1·8 g/d seemed to overcome the genetic differences, and therefore in supplementation trials, measuring these mutations may not be necessary		No in supplementation trials
Age	Plasma EPA and DHA is positively associated with age (approximately 38 % increase from age 20 to 79 years)	Erythrocyte EPA and DHA tended to be positively associated with age (approximately 19 % increase from age 20 to 75 years)		Yes
BMI	*n*-3 LCPUFA levels is negatively associated with erythrocyte EPA and DHA of 5·6 % or lower	No association with erythrocyte EPA and DHA >7 %		Yes
Girth	Inconclusive			No
Physical activity	Inconclusive			No
Alcohol	No or negative association between alcohol consumption and *n*-3 LCPUFA levels (except for wine)	Positive association between wine drinking and *n*-3 LCPUFA levels	The optimal amount of wine consumption seems to plateau between two and three glasses per d	Yes, but specified according to type and amount of alcohol
Smoking	Smoking is negatively associated with erythrocyte EPA and DHA	High intakes of *n*-3 LCPUFA seem to overcome this at least partially	Smoking is associated with a 6–17 % lower erythrocyte EPA and DHA	Yes
Bioavailability chemically-bound form	TAG form is better than EE form			Yes
Bioavailability krill oil *v.* fish oil	Inconclusive			No
Bioavailability ALA conversion to *n*-3 LCPUFA	ALA is converted to EPA but not DHA	Higher conversion of ALA to DHA in women compared with men		

FADS, fatty acid desaturase; GLA, *γ*-linolenic acid; AA, arachidonic acid; EE, ethyl ester; ALA, *α*-linolenic acid.

Whilst the focus of this systematic review was not to assess the effect of *n*-3 LCPUFA interventions, a few points regarding the importance of measuring *n*-3 LCPUFA levels pre- and post-supplementation are warranted. Assessing only dietary or supplemental intake of *n*-3 LCPUFA is not good enough to really demonstrate the efficacy of *n*-3 LCPUFA supplementation. For example, a study investigating the effect of 1·4 g/d of *n*-3 LCPUFA supplementation for 6 months in young people at ultra-high risk of psychotic disorders failed to show the efficacy of *n*-3 LCPUFA; but this was most likely due to the lack of compliance as more than half of the participants were non-compliant, and a limitation of this study was the lack of measuring the blood *n*-3 LCPUFA levels^(^ ^123^ ^)^. Another study measured blood *n*-3 LCPUFA levels pre- and post-supplementation where the participants consumed 1 g/d of *n*-3 LCPUFA through the consumption of *n*-3 LCPUFA-enriched foods, and this resulted in an increase in *n*-3 LCPUFA erythrocyte levels from 4 to 7·1 % of total erythrocyte fatty acids^(^ ^124^ ^)^. This increase in *n*-3 LCPUFA was associated with improvements in arterial compliance and chronic inflammation as assessed by serum C-reactive protein^(^ ^124^ ^)^, demonstrating the importance of measuring *n*-3 LCPUFA levels pre- and post-supplementation in terms of not only compliance but also assessing the effect of *n*-3 LCPUFA in relation to health outcomes. Furthermore, we recently reviewed the trials investigating the effect of *n*-3 LCPUFA supplementation in cardiac mortality and demonstrated that the dose of *n*-3 LCPUFA is important, but also ensuring that the study populations’ *n*-3 LCPUFA levels are not too high at baseline in order to alleviate a potential ceiling effect^(^ ^125^ ^)^. More recently two large clinical trials have been published, where the study using high dose (4 g/d, Reduction of Cardiovascular Events with IcosapentEthyl-Intervention Trial (REDUCE-IT)) showed efficacy in cardiovascular risk reduction^(^ ^126^ ^)^, and another study using a lower dose (1 g/d, VITamin D and omegA-3 triaL (VITAL)) did not show efficacy in the prevention of cardiovascular disease and cancer^(^ ^127^ ^)^. These trials further highlight the importance of dose of *n*-3 LCPUFA in clinical trials. Moreover, blood analyses of *n*-3 LCPUFA pre- and post-supplementation will (1) ensure the baseline levels are not too high to potentially reach a ceiling effect and (2) after supplementation show compliance to *n*-3 LCPUFA as well as being able to attribute the health outcomes to the effect of *n*-3 LCPUFA supplementation. Therefore, it is recommended that research into the health benefits of *n*-3 LCPUFA should include blood analyses of *n*-3 LCPUFA pre- and post-supplementation.

In conclusion, as summarised in Table 5 those scientifically supported factors that are associated with the *n*-3 LCPUFA levels must be considered in future (design of) studies. It is recommended that blood or tissue *n*-3 LCPUFA levels are measured in all types of research (including cross-sectional, cohort and clinical research), which assesses the health benefits of *n*-3 LCPUFA. Furthermore, in randomised controlled trials, *n*-3 LCPUFA levels should be measured pre- and post-supplementation. It is beyond the scope of this review to recommend which tissue or fraction of blood to measure, but there are a couple of good reviews available on this topic^(^ ^128^ ^,^ ^129^ ^)^.

## Supplementary material

For supplementary material accompanying this paper visit http://dx.doi.org/10.1017/S0007114519000138. click here to view supplementary material
