# Supplementary material for: Non-dietary factors associated with n-3 long-chain PUFA levels in humans – a systematic literature review
Source: Br J Nutr. 2019 Apr 14;121(7):793–808. doi: 10.1017/S0007114519000138 (PMC6521789; doi:10.1017/S0007114519000138)
Supplement: Supplementary file 1 [file S0007114519000138sup001.docx]

**Supplementary Material**

Supplemental table 1: Search Strategy

Word variations were searched. Key words were combined using connectors ‘AND’ & ‘OR’ and words were truncated when necessary to capture variations on the end of a word stem, e.g. metabol* to capture metabolic and metabolism.

| **Keyword** |  | **Alternative / related words** |
| --- | --- | --- |
|  |  |  |
| Keyword 1: Long chain polyunsaturated fatty acids | OR | ALTERNATIVES FOR KEYWORD 1: Docosahexaenoic acid (DHA) or Eicosapentaenoic acid (EPA) or n-3 polyunsaturated fatty acids or n-3 fatty acids or essential fatty acids or omega-3 or omega-3 fatty acids |
| **AND** | | |
| Keyword 2: "Age" OR "elderly" OR "old " OR “young” or "Sex" OR "gender" OR "gender” OR "genetics" OR "genotype" OR "FADS" OR "APOE" OR "gene expression" OR "ethnicity" OR "metabolism” OR “heritability” OR "smoking" OR "alcohol" OR " ETOH" OR "diet" OR "high fat diet" OR "high fat meal" OR "dietary fats" OR "fish intake" OR "fish servings" OR "vegetarian" OR "vitamin effect" OR "obesity" OR "weight" OR "body mass index" OR "BMI" OR "diabetes" OR "lifestyle" OR "food habits" OR "physical activity" OR “exercise” OR "environment" OR "education level" OR "determinants" OR "factors OR “influence" OR “effects” OR “content” OR “increase” OR “decrease” - Words variations searched and words truncated when necessary. | | |
| **OR** | | |
| KEYWORD 3 (SENTENCE): "factors influencing EPA and DHA" OR "factors influencing omega-3 fatty acid status" | | |

Supplemental table 2: Genetics Tables

Heritability

| Harris et al. 2012 ^(10)^  3196 subjects | Heritability  Also – Age, Gender, Cohort, Education, Blood Pressure, Heart rate, waist girth, smoking, lipids, FO supplementation, aspirin, other medications, BMI | Yes | Heritability explained 24% of the variance of the Omega-3 index. |
| --- | --- | --- | --- |

FADS

| Reference & subject numbers | Gene | Influence on EPA or DHA | Summary of main findings in relation to EPA and DHA |
| --- | --- | --- | --- |
| Gillingham et al. 2013 ^(26)^  26 (9 men and 17 women) | **FADS1 rs174545**  **FADS2 rs174583**  **FADS1 rs174561**  **FADS1 rs174537**  ELOVL2 rs953413 | Yes for EPA.  No for DHA.  Yes for AA | Homozygous minor allele carriers for each of the 4 SNPs had **↓** plasma EPA and AA composition compared to major allele subjects at baseline.  Homozygous minor allele carriers on the FXCO diet had increases in their plasma EPA composition to amounts higher than the control Western diet major allele carriers, suggesting ALA supplementation may be a useful way to increase EPA levels in homozygous minor allele carriers up to amounts comparable or even higher than the normal population.  No significant impacts for DHA were found. ELOVL2 was not associated with differences in plasma fatty acids. |
| Tintle et al. 2015 ^(27)^  2633 subjects | Analysis of over 2.5million SNPs for association with 14 different RBC fatty acids. Identified 191 different SNPs associated with at least 1 fatty acid. | No for EPA  No for DHA | The authors noted a negative (non significant) association between DHA and many of the SNPs, suggesting possible influence of genetic variability on n-3 LCPUFA, however results did not reach significance for EPA or DHA. |
| Malerba et al. 2008 ^(28)^  658 Cardiovascular patients | FADS1 rs174545, rs174556, rs174561  FADS1/FADS2 rs3834458, FADS2 rs174570, rs2524299, rs174583, rs174589, rs498793, rs174611  FADS2/FADS3 rs17831757, rs174627  FADS3 rs1000778 | No for EPA  No for DHA  Yes for AA | No association was found between minor or major allele carriers for EPA or DHA levels. Carriers of the minor alleles had significantly **↓** levels of AA. |
| Schaeffer et al. 2006 ^(29)^  727 Subjects | SNPs in the FADS1 and FADS2 gene region  FADS1 **rs174544,** rs174545, rs174546, **rs174553, rs174556, rs174561**  FADS2 **rs174568**, rs3834458, **rs968567, rs99780, rs174570, rs2072114, rs174583, rs174589,** rs175602, rs526126, rs174620, rs482548 | Yes for EPA.  No for DHA.  Yes for AA | Subjects carrying the minor alleles of the SNPs rs174544, rs174553, rs174556, rs174561, rs174568, rs968567, rs99780, rs174570, rs2072114, rs174583 and rs174589 exhibited **↓** levels of EPA.  DHA did not show any statistically significant association.  All haplotypes carrying minor alleles were also associated with **↓** EPA.  Subjects carrying minor alleles, (except rs526126 and rs482548) had **↓** levels of AA. |
| Rzehak et al. 2009 ^(30)^  535 subjects | FADS1 rs174556, rs174561  FADS2 rs3834458  (3 haplotypes determined) | No for EPA  No for DHA  Yes for AA | No significant associations determined for EPA or DHA. Whilst EPA showed no significant association, the trend was for lower EPA in minor allele carriers. This study does state that perhaps they did not have adequate power to identify significance in some PUFAs.  Carriers of the minor alleles had significantly **↓** levels of AA. |
| Tanaka et al. 2009 ^(31)^  InCHANTI = 1210 subjects  GOLDN = 1120 | **FADS1 rs174537**  ELOVL2 rs953413  HRH4 rs16940765,  SPARC rs17718324,  SLC26A10 rs2277324/rs923838 | Yes for EPA  No for DHA  Yes for AA | Presence of the minor allele (T) in rs174537 was associated with **↓** concentrations of EPA and AA, but no significant differences for DHA. |
| Baylin et al. 2007 ^(32)^  1694 case-control pairs | Common deletion FADS2 **rs3834458 *(TI-)*** | Yes for EPA  Yes for DHA in plasma  No for DHA in AT  Yes for AA | EPA and AA **↓** with the number of copies of the variant allele (1 or 2 deletions) in both adipose tissue and plasma fatty acids  DHA was not significantly different in AT (p=0.34), but in plasma it was significantly **↓** |
| Mathias et al. 2010 ^(25)^  224 subjects | rs174537 rs174545 rs174546 rs174553 rs174556 rs174561 rs174568 rs99780 rs174570 rs174575 rs2524299 rs174583 rs498793 rs174611 rs174627 rs1000778 | Yes for EPA  Yes for DHA  Yes for AA | In majority of the SNPs studied being a minor allele carrier was associated with **↓** EPA, DHA and AA |
| Al-Hilal et al. 2013 ^(24)^  310 subjects | FADS1 rs174537  FADS1 rs174561  FADS2 rs3834458 | Yes for EPA  Yes for DHA  Yes for AA | Subjects carrying minor allele of any of the three SNPs had significantly **↓** proportions of EPA, DHA and AA at baseline.  D5D and D6D activity had significantly **↓** estimated activities in minor allele carriers.  Response to supplementation was not different between minor and major carriers. |

ELOLV2

| Alsaleh et al. 2014 ^(33)^  310 women (aged 45 – 70 years) | **ELOLV2 rs3734398, rs2236212, rs953413** | Yes for DHA | Lower DHA levels at baseline in minor allele carriers and higher levels after supplementation in carriers versus non-carriers. |
| --- | --- | --- | --- |
| Tanaka et al. 2009 ^(31)^ | FADS1 rs174537  **ELOVL2 rs953413**  HRH4 rs16940765,  SPARC rs17718324,  SLC26A10 rs2277324/rs923838 | Yes for rs174537 for EPA.  Yes for rs953413 (ELOVL2) for EPA and DHA. | Presence of minor allele in rs953413 was associated with higher concentrations of EPA (in InCHANTi but not GOLDN) and **↓** concentrations of DHA (in both InCHANTI and GOLDN) |
| Gillingham et al. 2013 ^(26)^  26 (9 men and 17 women) | SNPs  FADS1 rs174545  FADS2 rs174583  FADS1 rs174561  FADS1 rs174537  **ELOVL2 rs953413** | No for EPA.  No for DHA. | ELOVL2 was not associated with differences in plasma fatty acids. |

ApoE4

| Samieri et al. 2011 ^(38)^  1135 subjects | ApoE4 allele carriers | No for EPA No for DHA | No differences between carriers and non carriers in whole sample. |
| --- | --- | --- | --- |
| Yassine et al. 2016 ^(36)^  384 subjects | ApoE4 allele in Alzheimer’s disease | No | No difference in baseline or end of intervention plasma DHA levels for carriers or non carriers. |
| Chouinard-Watkins et al. 2013 ^(35)^  40 subjects | ApoE4 allele carriers verse non carriers in elderly (>50yrs) | No for baseline plasma levels. | Baseline plasma levels were not different between groups – note however very small group, probably not statistically powerful enough to pick up baseline differences. |
| Whalley et al. 2008 ^(37)^  113 subjects | ApoE4 carriers verse non carriers in elderly (>62yrs) | No | Snap shot erythrocyte DHA and EPA levels did not differ between groups. Cognitive performance was better in participants with higher levels of n-3 LCPUFA in the non E4 carriers only. No impact of higher n-3 LCPUFA on cognitive health of E4 carriers. |
| Plourde et al. 2009 ^(34)^  28 men | L162V polymorphism and ApoE4 allele | Yes for ApoE4.  No for L162V | E4 carriers had higher plasma TAG EPA and DHA compared to non carriers at baseline. |

Supplemental table 3: Age Tables

| Reference | Biomarkers reported | Expression of data | Age Groups | EPA | | | | DHA | | |
| --- | --- | --- | --- | --- | --- | --- | --- | --- | --- | --- |
| Plourde et al. 2011 ^(43)^ | Plasma Fatty Acids | mean % | 27 years (6 subjects)  77 years (6 subjects) | 0.6  1.2  P=0.012 | | | | 1.7  1.4  P=0.872 | | |
| Vandal et al. 2008 ^(42)^ | Plasma Fatty Acids | mean % | 18 – 29 (9 subjects)  70 – 79 (10 subjects) | 0.6  1.0  P<0.05 | | | | 1.5  1.6  ns | | |
| Rees et al. 2006 ^(41)^  Placebo group at Baseline | Plasma Fatty Acids | % by wt of total fatty acids | 18 – 42  53 – 70 | 0.9  1.5  P<0.05 | | | | 3.1  5.0  P<0.05 | | |
| Rees et al. 2006 ^(41)^  Low EPA group at Baseline | Plasma Fatty Acids | % by wt of total fatty acids | 18 – 42  53 – 70 | 1.2  1.5  P<0.05 | | | | 3.4  4.8  P<0.05 | | |
| Rees et al. 2006 ^(41)^  Moderate EPA group at Baseline | Plasma Fatty Acids | % by wt of total fatty acids | 18 – 42  53 - 70 | 0.9  1.3  P<0.05 | | | | 3.3  4.3  P<0.05 | | |
| Rees et al. 2006 ^(41)^  High EPA group at Baseline | Plasma Fatty Acids | % by wt of total fatty acids | 18 – 42  53 – 70 | 1.2  1.3  P<0.05 | | | | 3.3  4.6  P<0.05 | | |
| Fortier et al. 2010 ^(40)^ | Plasma Fatty Acids | mean % | 18 – 30  >65 years | 0.5  1.0  P<0.05 | | | | 1.4  1.7  ns | | |
| Hennebelle et al. 2016 ^(44)^ | Plasma Fatty Acids | mg/L | 25±2.8 years (10 subjects)  73±7.0 years (10 subjects) | 20  40  P<0.05 | |  | | 47  63  ns | |  |
| Walker et al. 2014 ^(39)^ | RBC Fatty Acids | % by wt of total fatty acids | 20 – 39 (n=66)  40 – 59 (n=68)  60 -79 (n=69) | 1.86  1.89  2.60  ns | | | | 5.24  5.20  5.43  ns | | |
| Sfar et al. 2010 ^(54)^ | Plasma Fatty Acids | mean % | 42 – 59  60 - 82 | Women  1.17  0.93  ns | | Men  0.92  0.73  ns | | Women  3.51  3.02  sig | | Men  2.88  2.89  ns |
| Dewailly et al. 2002 ^(58)^ | Plasma Fatty Acids | % by wt of total fatty acids | 18 – 34  35 – 49  50 - 74 | 0.5  0.74  1.23  P=0.0001 | | | | 2.4  3.03  4.01  P=0.0001 | | |
| Dewailly et al. 2001 ^(59)^ | Plasma Fatty Acids | % by wt of total fatty acids | 18 – 34  35 – 49  50 - 74 | 0.44  0.49  0.50  P=0.0001 | | | | 1.09  1.23  1.30  P=0.0001 | | |
| Dewailly et al. 2001 ^(61)^  (Inuit population) | Plasma Fatty Acids | % by wt of total fatty acids | 18 -39  >40 years | 1.5  3.58  P=0.0001 | | | | 4.03  5.77  P=0.0001 | | |
| Kuriki et al. 2002 ^(46)^ | Plasma Fatty Acids | % by wt of total fatty acids | 32 – 42  43 – 50  51 - 66 | 2.32  2.61  2.88  ns | | | | 5.3  5.42  5.55  ns | | |
| Bjerve et al. 1989 ^(45)^ | Plasma Fatty Acids | mgl-1 | 20 – 29  30 – 39  40 -49 | 15.2  25.3  36.1 |  | | | No data available | |  |
| Kawabata et al. 2011 ^(53)^ | Plasma Fatty Acids  Plasma TAG  Plasma CE  Plasma PL  Erythrocyte PL | % by wt of total fatty acids  % by wt of total fatty acids  % by wt of total fatty acids  % by wt of total fatty acids | Women 20 years / Men 20 years  Women 56 – 73 / Men 60 – 75  Women 20 years / Men 20 years  Women 56 – 73 / Men 60 – 75  Women 20 years / Men 20 years  Women 56 – 73 / Men 60 – 75  Women 20 years / Men 20 years  Women 56 – 73 / Men 60 – 75 | Women  0.3  0.5  ns  1.4  1.3  ns  2.2  2.3  ns  1.4  1.9  ns | Men  0.2  1.5  P<0.001  0.6  2.7  P<0.001  1.1  3.8  P<0.001  0.8  2.3  P<0.001 | | | Women  1.4  1.4  ns  0.7  0.9  P<0.001  5.5  4.9  P<0.05  6.1  6.4  ns | | Men  1.0  4.1  P<0.001  0.6  1.1  P<0.001  5.5  6.6  P<0.01  6.0  6.7  P<0.001 |
| Babin et al. 1999 ^(57)^ | Plasma Fatty Acids  RBC | % by wt of total fatty acids  % by wt of total fatty acids | 20 – 48  >75  20 – 48  >75 | 0.77  0.81  ns  0.64  0.60  ns | | | | 1.98  1.88  ns  3.18  2.69  ns | | |
| de Groot et al. 2009 ^(50)^ | Plasma PL | Values not given | 36 – 88 years | Plasma phospholipid concentrations of EPA and DHA significantly **↑** in older individuals  Age explained 3.9% and 2.3% of the variance in EPA and DHA, respectively | | | | | | |
| Ogura et al. 2010 ^(47)^ | Plasma PL  RBC PL  Adipose Tissue | Values not given | 27 – 81 years | Significant positive correlation between EPA + DHA in plasma and erythrocyte PL fractions and age.  No correlation between Adipose TAG EPA + DHA fractions and age. | | | | | | |
| Crowe et al. 2008 ^(55)^ | Serum Fatty Acids  PL  CE  TAG | mol%  mol%  mol% | 15 – 24  25 – 44  45 – 64  65+  15 – 24  25 – 44  45 – 64  65+  15 – 24  25 – 44  45 – 64  65+ | Women  0.78  0.93  1.08  1.14  P<0.001  0.77  0.94  1.12  1.22  P<0.001  0.20  0.26  0.28  0.29  P<0.001 | Men  0.86  1.07  1.14  1.12  P<0.001  0.88  1.09  1.21  1.17  P<0.001  0.22  0.28  0.29  0.25  P=0.036 | | | Women  2.58  2.67  2.81  2.79  P<0.001  0.50  0.51  0.54  0.54  P=0.097  0.49  0.53  0.52  0.52  P=0.795 | | Men  2.29  2.55  2.69  2.67  P=0.035  0.44  0.50  0.51  0.52  P<0.001  0.42  0.52  0.51  0.47  P=0.140 |
| Otsuka et al. 2013 ^(48)^ | Serum Fatty Acids | mmol/L | 40 – 49  50 – 59  60 – 69  70 - 79 | Women  164  259  278  290  P<0.0001 | Men  203  274  319  386  P<0.0001 | | | Women  408  513  573  600  P<0.0001 | | Men  454  542  581  561  P<0.0001 |
| Tavendale et al. 1992 ^(62)^ | Adipose Tissue Fatty Acids | Mean % | 40 – 44 years  45 – 49 years  50 – 54 years  55 – 59 years |  | | | Women  0.18  0.19  0.23  0.25  P<0.001 | | Men  0.18  0.19  0.19  0.19  P<0.001 | |
| Bolton-Smith et al. 1997 ^(51)^ | Adipose Tissue Fatty Acids | Values not given | 40 – 59 years | Adipose Tissue Fatty acid levels of DHA + DPA **↑** with increasing age. | | | | | | |
| Harris et al. 2013 ^(23)^ | RBC Fatty Acids | Values not given | 11 – 90 years | EPA **↑** 13% per decade up to 70years  DHA **↑** 6% per decade  Omega-3 index **↑** 7% per decade up to 70years  EPA **↓** 9% per decade after age 70years | | | | | | |
| Block et al. 2008 ^(52)^ | RBC EPA + DHA (Omega-3 Index) | Values not given | 62 ± 12 years | Age **↑** Omega-3 index by 5.3% for a 10 year increase | | | | | | |
| Sands et al. 2005 ^(49)^ | RBC EPA + DHA (Omega-3 Index) | Values not given | 20 – 80 years | Every additional 10 years of age **↑** EPA + DHA by 0.5units | | | | | | |
| Itomura et al. 2008 ^(60)^ | RBC EPA + DHA (Omega-3 Index) | Values not given | 18 – 70 years | RBC total phospholipid EPA + DHA composition **↑** with increasing age | | | | | | |
| Saga et al. 2012 ^(56)^ | Whole Blood | Values not given | 10 – 80 years | Proportions of EPA + DHA in whole blood **↑** with age  This result was the same for both fish oil supplement users and non-users. | | | | | | |
| Harris et al. 2012 ^(10)^ | RBC EPA + DHA (Omega-3 Index) | Values not given |  | Age **↑** Omega-3 index by 5% for a 10 year increase | | | | | | |
